# Effects of Different Rearing Systems (Cage vs. Free-Range) on Growth Performance, Serum Biochemical Parameters, Slaughter Performance, Cecal Microbiota, and Hepatic Metabolism of Yellow-Feathered Broilers

**DOI:** 10.3390/ani16121920

**Published:** 2026-06-21

**Authors:** Xiaohang Nie, Jiasheng Li, Yuanyuan Cui, Jiang Yuan, Fengming Li, Yong Chen, Jiancheng Liu

**Affiliations:** 1 Research Center for Biological Feed and Animal Gut Health, College of Animal Science, Xinjiang Agricultural University, Urumqi 830052, China; 17630848120@163.com (X.N.); ljs1693095665@163.com (J.L.); lifming@163.com (F.L.); xjaucy@163.com (Y.C.); 2 Xinjiang Tycoon Group Co., Ltd., Changji 831199, China; 18140910355@163.com (Y.C.); 18199843513@163.com (J.Y.)

**Keywords:** cage rearing, free-range rearing, yellow-feathered broilers, gut microbiota, hepatic metabolism

## Abstract

This experiment investigated the effects of two rearing systems, cage and free-range, on growth performance, serum biochemical parameters, slaughter performance, cecal microbiota, and hepatic metabolism of yellow-feathered broilers. A total of 240 healthy 21-day-old Liangfenghua yellow-feathered male broilers with similar body weight were randomly assigned to a cage group (LY) and a free-range group (SY), with 10 replicates per group and 12 birds per replicate. All birds were fed the same diet until 63 days of age. Compared with the LY group, the SY group had significantly lower final body weight, average daily gain, and abdominal fat percentage (*p* < 0.05), while average daily feed intake and feed-to-gain-ratio were significantly higher (*p* < 0.05). The Shannon and Simpson indices of the cecal microbiota were significantly higher in the SY group (*p* < 0.05), and the genera *Bacteroides*, *Lactobacillus*, *Rikenella*, and *Oscillibacter* were specifically enriched. A total of 560 differential metabolites were identified by liver non-targeted metabolomics, and these metabolites were significantly enriched in the necroptosis, cysteine and methionine metabolism, thiamine metabolism, and amino sugar and nucleotide sugar metabolism pathways (*p* < 0.05). Correlation analysis between cecal microbiota and liver metabolites revealed that the differentially abundant bacterial genera showed significant negative correlations with multiple amino acid metabolites in the liver. In conclusion, the free-range rearing system reduced the growth performance of yellow-feathered broilers but enriched specific bacterial genera, increased gut microbiota diversity, and modulated host amino acid metabolism and energy homeostasis through the “gut microbiota–liver” axis, ultimately inducing an adaptive metabolic state characterized by reduced abdominal fat deposition and remodeling of hepatic metabolic pathways.

## Simple Summary

Consumers are increasingly concerned about how chickens are raised, yet the biological effects of different rearing systems remain poorly understood. This study examined whether raising chickens in cages or in a free-range environment affects their growth, gut health, and liver metabolism. We raised 240 yellow-feathered male broilers under both systems, fed them the same diet, and then measured their growth performance, blood indicators, gut microbes, and liver metabolites. The results showed that free-range chickens grew more slowly and deposited less abdominal fat than caged chickens. Additionally, free-range chickens had a more diverse community of gut bacteria, with greater numbers of beneficial microbes, and showed clear changes in liver metabolic pathways. These changes in gut bacteria were closely linked to altered levels of certain amino acid-related compounds in the liver. These findings suggest that the free-range environment, although less efficient for rapid growth, promotes a healthier gut and a metabolic state that may help chickens better cope with environmental challenges. This work provides new scientific insights that could guide farming practices aimed at improving both chicken health and meat quality.

## 1. Introduction

As global chicken consumption rises, broiler production has grown rapidly over the past few decades [1]. According to the Food and Agriculture Organization of the United Nations (FAO), global chicken meat production reached 128 million metric tons in 2024, making it the world’s leading meat product [2]. China’s broiler industry is characterized by large-scale production, diverse breeds, and high output. In 2024, China produced 14.842 billion broilers, of which fast-growing white-feathered broilers accounted for 11.508 billion birds (77.54%) and slow-growing yellow-feathered broilers accounted for 3.334 billion birds (22.46%) [3]. White-feathered broilers exhibit rapid growth, high feed conversion efficiency, tender meat, and low cost; however, their relatively short growth cycle results in less rich flavor in the meat. In contrast, yellow-feathered broilers require a longer rearing period, which contributes to superior flavor and more nutritious meat, and thus they are preferred by consumers [4,5]. The production of high-quality meat products is closely associated with animal breed, rearing system, animal welfare, and production procedures [6].

The rearing system is an important non-genetic factor influencing broiler production and welfare [7]. It is affected by geographical environment [8], broiler breed [9], and policy regulations [10], and in turn influences broiler growth performance [11], health status [12], and product quality [13]. Currently, cage and free-range systems are the two main rearing systems for broiler production worldwide [14]. The cage system was developed to meet the demands of modern large-scale, industrialized, and standardized production. It maximizes space utilization, enables standardized feeding management, and facilitates effective disease prevention, thereby achieving higher productivity and a better feed conversion ratio [11]. However, this industrialized production can adversely affect broiler health. The contradiction between high growth rate and unbalanced body development can lead to various diseases, such as sudden death syndrome, ascites, breast blisters, and leg bone degeneration [15]. Moreover, the cage system restricts the expression of natural behaviors and does not comply with animal welfare principles [16].

In contrast, the free-range system emphasizes free movement and growth in a natural environment, allowing birds greater opportunities for natural behaviors such as walking, running, foraging, and dust bathing [17]. It also yields meat of superior quality and higher levels of essential nutrients, such as amino acids and organic acids [18]. Therefore, the free-range system offers advantages in improving animal welfare and chicken meat quality [19]. However, compared with the cage system, the free-range environment is more complex and exerts a more pronounced impact on animal health. Dikmen and Gündüz [20] compared the effects of cage and floor litter free-range systems on the performance, welfare, and behavioral parameters of Ross 308 broilers, and found that the floor litter free-range system significantly improved broiler performance, welfare, and behavioral indicators. Xu et al. [21] compared the effects of cage and free-range systems on the growth performance, slaughter performance, and meat quality of Wenchang chickens, and found that the cage system enhanced growth performance and slaughter performance, while the free-range system improved blood lipid metabolism, meat quality, and flavor compound content. Zhang et al. [4] investigated the effects of floor free-range and cage rearing systems and sex on the growth performance, meat quality, and gut microbiota of yellow-feathered broilers, and found that caged males exhibited the best growth performance, a result that may be attributed to differences in gut microbiota between the cage and floor free-range systems. Li et al. [22] and Zeng et al. [12] studied the effects of cage and free-range rearing systems on the intestinal morphology, intestinal metabolites, and gut microbiota and their metabolites in Lüeyang black-boned chickens. They found that the rearing system significantly affected intestinal morphology, the composition and function of the gut microbiota, and intestinal metabolites, thereby altering nutrient metabolism, host adaptive immunity, and muscle development and quality, suggesting that the rearing system could potentially influence broiler health and performance through the gut–liver axis [23]. However, studies that use gut microbiota and liver metabolomics to elucidate how animals respond to rearing environmental stress and regulate their physiological state, and to reveal the mechanisms by which different rearing systems (cage vs. free-range) affect the animal body, remain scarce.

Therefore, using yellow-feathered broilers as the experimental model, this study investigated the effects of two rearing systems (cage vs. free-range) on growth performance, serum biochemical parameters, gut microbiota, and hepatic metabolism. The aim was to elucidate the mechanisms underlying host–environment interactions, providing a theoretical basis for healthy broiler production and a scientific foundation for improving flavor formation and nutritional quality in free-range chicken meat.

## 2. Materials and Methods

### 2.1. Ethical Statement

All animal experiments were conducted in accordance with the relevant national guidelines and approved by the Animal Welfare and Care Use Committee of Xinjiang Agricultural University (Xinjiang, China) (protocol number: 2024005).

### 2.2. Experimental Design and Rearing Management

A total of 300 healthy, one-day-old Liangfenghua yellow-feathered male broilers were brooded together until 21 days of age. From these, 240 healthy male broilers with similar body weights were selected and randomly assigned to a cage group (LY group) and a free-range group (SY group), each with 10 replicates of 12 birds. All birds were fed the same diet until 63 days of age. The diet was formulated in accordance with NY/T 3645-2020 Nutrient Requirements of Yellow-Feathered Broilers, and its composition and nutrient levels are presented in Table 1. Birds in the LY group were reared in a modern temperature-controlled chicken house in three-tier, partitioned cages (12 birds per partition, stocking density of 0.0625 m^2^/bird, 25 °C). Birds in the SY group were reared in a free-range system in a natural environment adjacent to the chicken house of the LY group (12 birds per pen, stocking density of 1.25 m^2^/bird). All experimental birds had ad libitum access to the same feed and water, and the SY group did not have access to any edible materials such as weeds or their seeds to avoid confounding effects caused by differences in nutrient intake, thereby enabling the evaluation of differences arising from the rearing environment (air quality, stocking density, light type, temperature variation, etc.). The LY group was illuminated with LED lights, and the photoperiod was synchronized with the natural photoperiod of the SY group. Immunization and other routine management procedures were conducted in accordance with NY/T 1871-2010 Technical Regulations for Feeding and Management of Yellow-Feathered Broilers.

### 2.3. Sample Collection and Measurement of Indicators

#### 2.3.1. Growth Performance

During the experimental period, feed intake was recorded per-replicate for each group. At 21 and 63 days of age, birds were weighed per replicate after a 12-h fast. Average daily feed intake (ADFI), average daily gain (ADG), and feed-to-gain ratio (F/G) were calculated for each group.

#### 2.3.2. Serum Biochemical Parameters

At 64 days of age, one bird with a body weight near the replicate average was randomly selected from each replicate. Blood was collected from the wing vein, and serum was separated. Serum levels of alanine aminotransferase (ALT), aspartate aminotransferase (AST), total protein (TP), albumin (ALB), globulin (GLB), triglycerides (TG), total cholesterol (T-CHO), high-density lipoprotein cholesterol (HDL-c), low-density lipoprotein cholesterol (LDL-c), glucose (GLU), and blood urea nitrogen (BUN) were measured using a TBA-FX8 automatic biochemical analyzer (Canon Medical Systems Corporation, Ota, Tokyo, Japan).

#### 2.3.3. Slaughter Performance

At 64 days of age, one bird with a body weight close to the replicate average was randomly selected from each replicate, weighed, and slaughtered by jugular vein exsanguination. Slaughter was performed in accordance with Terminology and Measurement Methods for Poultry Growth Performance (NY/T 823-2020). Live body weight, carcass weight, eviscerated weight, semi-eviscerated weight, breast muscle weight, leg muscle weight, and abdominal fat weight were measured. Dressing percentage, eviscerated percentage, semi-eviscerated percentage, breast muscle percentage, leg muscle percentage, and abdominal fat percentage were calculated.

#### 2.3.4. Cecal Microbiota 16S rDNA Amplicon Sequencing

After slaughter, one chicken with a body weight close to the group average was selected from each of the five replicates per group. Cecal contents were rapidly collected, placed in 2 mL sterile cryovials, snap-frozen in liquid nitrogen, and stored at −80 °C. Total microbial DNA was extracted from the cecal contents using the CTAB method. The bacterial 16S rRNA gene V3–V4 hypervariable region was amplified with the universal primers: 515F (5′-CCTAYGGGRBGCASCAG-3′) and 806R (5′-GGACTACNNGGGTATCTAAT-3′). The amplification program consisted of an initial denaturation at 98 °C for 1 min; 30 cycles of denaturation at 98 °C for 10 s, annealing at 50 °C for 30 s, and extension at 72 °C for 30 s; and a final extension at 72 °C for 5 min. Sequencing libraries were prepared using the TruSeq^®^ DNA PCR-Free Sample Preparation Kit (Illumina, Inc., San Diego, CA, USA). After passing quality control, the libraries were sequenced on a NovaSeq 6000 platform (Illumina, Inc., San Diego, CA, USA). Raw data were filtered, and chimeras were removed to obtain high-quality sequences. The sequencing was performed by Beijing Novogene Biotechnology Co., Ltd. (Beijing, China).

#### 2.3.5. Liver Untargeted Metabolomics Profiling

After slaughter, one chicken with a body weight close to the group average was selected from each of the five replicates per group. Liver samples were collected from the same anatomical location, immediately snap-frozen in liquid nitrogen, and stored at −80 °C. A 100 mg aliquot of liquid nitrogen-ground liver tissue was weighed into an EP tube, and 500 μL of 80% methanol was added. The mixture was vortexed, placed on ice for 5 min, and then centrifuged at 15,000× *g* for 20 min at 4 °C. An aliquot of the supernatant was diluted with water to a final methanol concentration of 53%, then centrifuged again at 15,000× *g* for 20 min at 4 °C. The resulting supernatant was collected for LC-MS analysis. Chromatographic separation was performed on a Hypersil Gold C18 column at 40 °C with a flow rate of 0.2 mL/min. The mobile phase consisted of 0.1% formic acid in water (A) and methanol (B). The mass spectrometric scan range was set to m/z 100–1500. The ESI source parameters were as follows: spray voltage, 3.5 kV; sheath gas flow rate, 35 psi; auxiliary gas flow rate, 10 L/min; ion transfer tube temperature, 320 °C; ion injection RF level, 60; and auxiliary gas heater temperature, 350 °C. Data were acquired in both positive and negative polarity modes, and MS/MS spectra were obtained using data-dependent acquisition. Data were statistically analyzed on the cloud platform of Novogene Biotechnology Co., Ltd., Beijing, China. (Available online: https://magic-plus.novogene.com (accessed on 20 March 2026)).

### 2.4. Statistical Analysis

Preliminary data organization and calculation were performed Microsoft Excel 2021. Statistical analysis was conducted in IBM SPSS Statistics 27 (IBM Corp., Armonk, NY, USA) using an independent samples *t*-test. Results are expressed as mean ± standard deviation (SD). A probability value of *p* < 0.05 was considered statistically significant, and *p* > 0.05 was considered non-significant.

## 3. Results

### 3.1. Effects of Different Rearing Systems on Growth Performance of Yellow-Feathered Broilers

As shown in Table 2, compared with the SY group, the LY group had significantly higher body weight at 63 days of age and average daily gain (ADG), and significantly lower average daily feed intake (ADFI) and feed-to-gain ratio (F/G) (*p* < 0.05).

### 3.2. Effects of Different Rearing Systems on Serum Biochemical Parameters of Yellow-Feathered Broilers

As shown in Table 3, no significant differences were observed between the LY and SY groups in serum levels of total protein (TP), albumin (ALB), globulin (GLB), blood urea nitrogen (BUN), triglycerides (TG), total cholesterol (T-CHO), high-density lipoprotein cholesterol (HDL-c), low-density lipoprotein cholesterol (LDL-c), alanine aminotransferase (ALT), aspartate aminotransferase (AST), or glucose (GLU) (*p* > 0.05).

### 3.3. Effects of Different Rearing Systems on Slaughter Performance of Yellow-Feathered Broilers

As shown in Table 4, the SY group had a significantly lower abdominal fat percentage than the LY group (*p* < 0.05). No significant differences were observed between the two groups in dressing percentage, semi-eviscerated percentage, eviscerated percentage, breast muscle percentage, or leg muscle percentage (*p* > 0.05).

### 3.4. Effects of Different Rearing Systems on Cecal Microbiota of Yellow-Feathered Broilers

#### 3.4.1. ASV Analysis of Cecal Microbiota Under Different Rearing Systems

**Figure 1:**
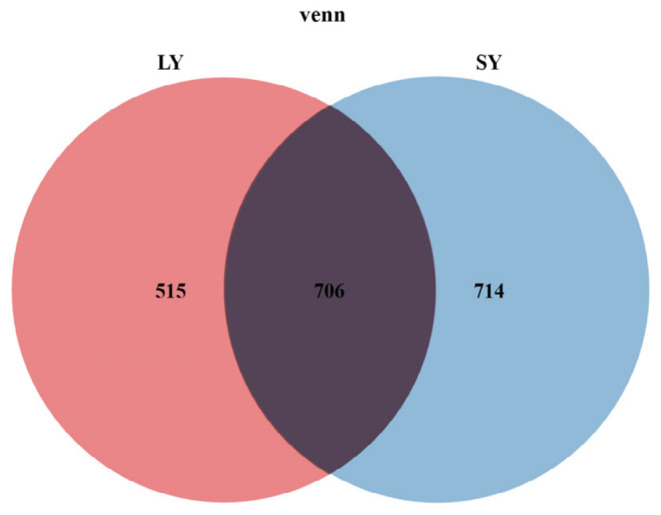
Venn diagram of ASVs in the cecal microbiota of yellow-feathered broilers under different rearing systems.

A Venn diagram was constructed from the amplicon sequence variants (ASVs) obtained (Figure 1). As shown in the figure, a total of 1935 ASVs were identified in the cecal microbiota of the LY and SY groups. Of these, 706 ASVs were shared by both groups, while 515 ASVs were unique to the LY group and 714 ASVs were unique to the SY group.

#### 3.4.2. Diversity Analysis of Cecal Microbiota Under Different Rearing Systems

**Figure 2:**
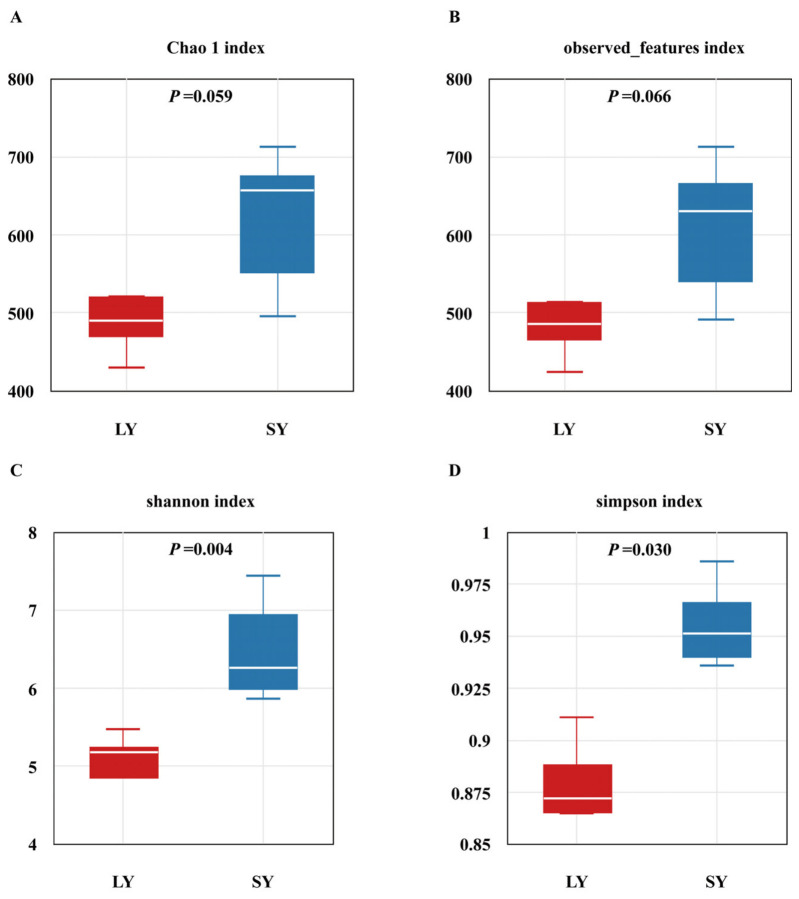
Alpha diversity analysis of the cecal microbiota of yellow-feathered broilers under different rearing systems. (**A**) Chao1 index; (**B**) Observed species; (**C**) Shannon index; (**D**) Simpson index.

Figure 2 shows the alpha diversity of the cecal microbiota in yellow-feathered broilers reared in cage versus free-range systems. The Shannon and Simpson indices were significantly higher in the SY group than in the LY group (*p* < 0.05). The Chao1 index was also higher in the SY group, but the difference was not statistically significant (*p* > 0.05).

**Figure 3:**
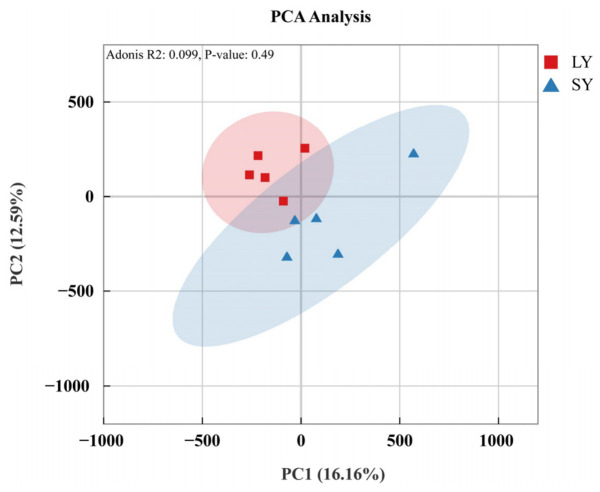
Beta diversity analysis of the cecal microbiota of yellow-feathered broilers under different rearing systems (PCA plot).

The beta diversity analysis of the cecal microbiota is shown in Figure 3. Principal component analysis (PCA) revealed that the first two principal components accounted for 16.16% and 12.59% of the total variance, respectively, and the scatter plots of the two groups showed partial overlap. PERMANOVA (Adonis) indicated no significant difference in microbial community structure between the two groups (*p* > 0.05).

#### 3.4.3. Effects of Different Rearing Systems on the Composition and Relative Abundance of Cecal Microbiota in Yellow-Feathered Broilers

**Figure 4:**
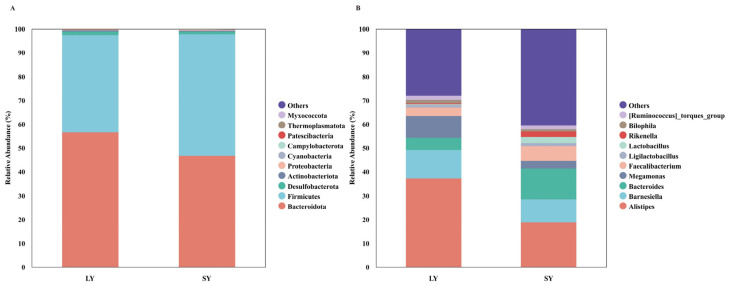
Cecal microbiota composition of yellow-feathered broilers under different rearing systems. (**A**) Phylum level; (**B**) Genus level.

The dominant phyla of the cecal microbiota under the two rearing systems are presented in Figure 4A and Table S1. As shown in Figure 4A, the dominant phyla common to both groups were *Bacteroidota*, *Firmicutes*, *Desulfobacterota*, *Actinobacteriota*, *Proteobacteria*, *Cyanobacteria*, and *Campylobacterota*. In the LY group, *Bacteroidota* was the predominant phylum (56.42%), followed by *Firmicutes* (41.05%), In contrast, *Firmicutes* was the most abundant phylum in the SY group, (51.18%), followed by *Bacteroidota* (46.68%). Compared with the LY group, the relative abundance of *Firmicutes* slightly increased, while that of *Bacteroidota* slightly decreased in the SY group. Moreover, the relative abundance of Cyanobacteria was significantly higher in the SY group than in the LY group (*p* < 0.05).

At the genus level, the top 10 most abundant genera in the cecal contents are shown in Figure 4B and Table S2. These genera were *Alistipes*, *Barnesiella*, *Bacteroides*, *Megamonas*, *Faecalibacterium*, *Ligilactobacillus*, *Lactobacillus*, *Rikenella*, *Bilophila*, and the *[Ruminococcus] torques* group. Compared with the LY group, the SY group had significantly higher relative abundances of *Lactobacillus* and *Rikenella* (*p* < 0.05), a tendency toward increased *Bacteroides* abundance (*p* = 0.064), and a tendency toward decreased *Alistipes* abundance (*p* = 0.058).

**Figure 5:**
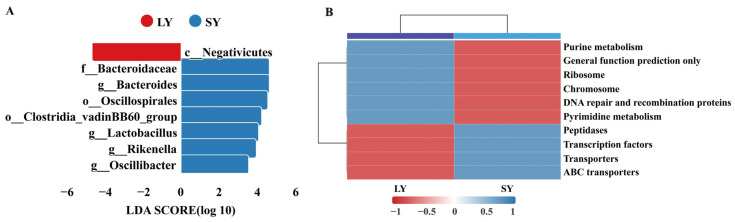
LEfSe analysis (LDA score = 3.5) and PICRUSt-predicted functional clustering heatmap of relative abundance of the cecal microbiota in yellow-feathered broilers under different rearing systems. (**A**) LEfSe analysis; (**B**) Clustering heatmap of PICRUSt functional prediction.

Biomarker taxa between the two groups were identified by LEfSe analysis (Figure 5A). At the genus level, *Bacteroides*, *Lactobacillus*, *Rikenella*, and *Oscillibacter* were significantly enriched in the SY group. Differential microbial metabolic pathways were functionally predicted using PICRUSt (Figure 5B). Compared with the LY group, the SY group showed downregulation of purine metabolism, general function prediction only, ribosome, chromosome, DNA repair and recombination proteins, and pyrimidine metabolism, pathways generally associated with cell growth, division, genetic information transfer, and maintenance. Conversely, peptidases, transcription factors, transporters, and ABC transporters were upregulated. These pathways are typically associated with environmental adaptation, nutrient acquisition, and stress responses.

### 3.5. Effects of Different Rearing Systems on Hepatic Metabolism of Yellow-Feathered Broilers

#### 3.5.1. Principal Component Analysis (PCA)

**Figure 6:**
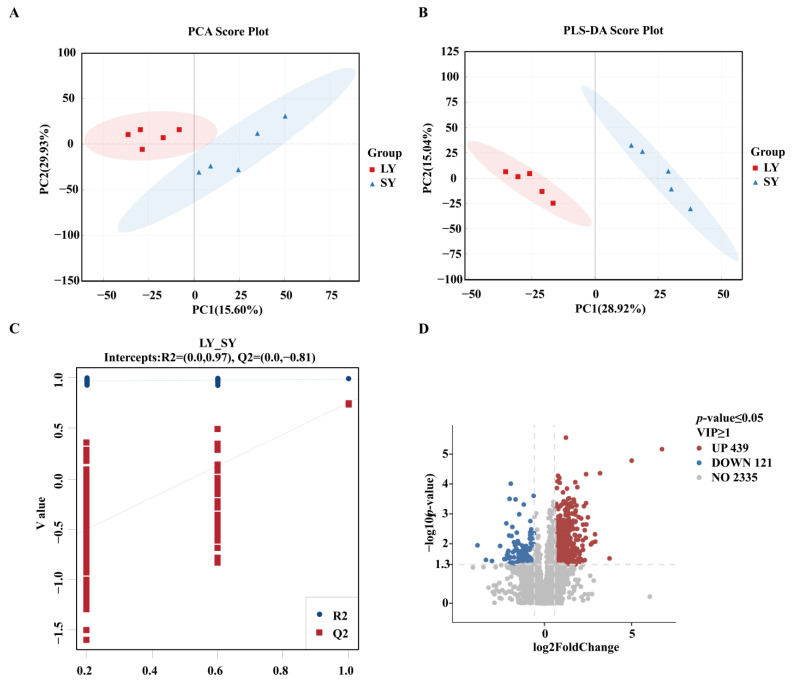
(**A**) Principal component analysis (PCA) score plot of liver metabolites in yellow-feathered broilers under different rearing systems; (**B**) Partial least squares discriminant analysis (PLS-DA) score plot; (**C**) Permutation test of the PLS-DA model; (**D**) Volcano plot showing differential liver metabolites between the two rearing systems.

The PCA of hepatic metabolites from yellow-feathered broilers under the cage and free-range rearing systems is shown in Figure 6A. Within each group, samples clustered tightly, and a clear separation between the two groups was evident, indicating that the hepatic metabolite profiles differed markedly between the two groups.

#### 3.5.2. Partial Least Squares-Discriminant Analysis (PLS-DA)

In this study, the PLS-DA model yielded R^2^Y = 0.99 and Q^2^Y = 0.75, both exceeding 0.5, indicating that the model had high explanatory power and predictive ability. As shown in Figure 6B, clear separation between the two groups was observed, effectively distinguishing the LY and SY group samples.

To further verify whether the PLS-DA model was overfitted, a 200-permutation test was performed. According to the permutation test plot (Figure 6C), the Q2 intercept was −0.81, which is less than zero. These results indicate that the model was not overfitted, confirming that the obtained data are reliable and can be used for subsequent differential metabolite screening.

#### 3.5.3. Analysis of Differential Hepatic Metabolites Under Different Rearing Systems

A total of 2895 metabolites were identified in the livers of yellow-feathered broilers reared under the cage versus free-range systems using non-targeted LC-MS/MS analysis. Based on the criteria of PLS-DA VIP > 1, fold change (FC) > 1.5 or FC < 0.667, and *p* < 0.05, 560 differential metabolites were identified, including 439 upregulated and 121 downregulated metabolites (Supplementary Tables S3 and S4). There differential metabolites were visualized and are presented as a volcano plot in Figure 6D.

#### 3.5.4. Functional Enrichment Analysis of Differential Hepatic Metabolites Under Different Rearing Systems

**Figure 7:**
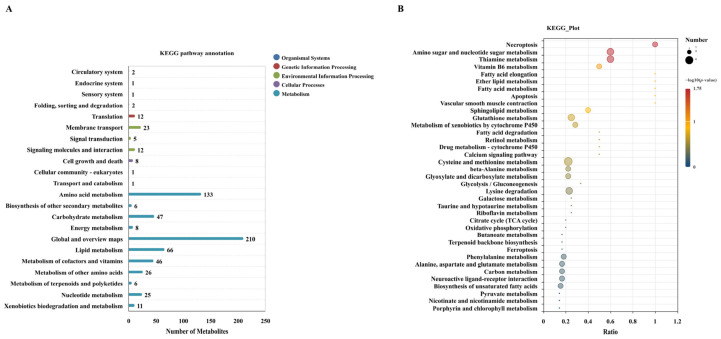
KEGG pathway annotation and bubble plot of differential liver metabolites under different rearing systems. (**A**) Pathway annotation diagram; (**B**) Bubble plot.

Under cage versus free-range rearing systems, the differential hepatic metabolites of yellow-feathered broilers were collectively enriched in 22 metabolic pathways (Figure 7A), which were mainly concentrated in the categories of Global and overview maps, Amino acid metabolism, Lipid metabolism, Carbohydrate metabolism, and Metabolism of cofactors and vitamins. KEGG pathway enrichment analysis (Figure 7B) showed that the differential metabolites between the cage and free-range groups were significantly enriched in necroptosis (*p* = 0.018), thiamine metabolism (*p* = 0.019), and amino sugar and nucleotide sugar metabolism (*p* = 0.019).

### 3.6. Correlation Analysis Between Cecal Microbiota and Hepatic Metabolites in Yellow-Feathered Broilers Under Different Rearing Systems

**Figure 8:**
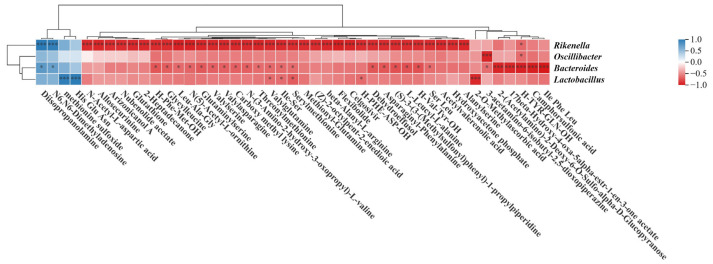
Correlation heatmap between differential bacterial genera in the cecal microbiota and differential hepatic metabolites of yellow-feathered broilers under different rearing systems. Note: Rows represent bacterial genera with LDA score > 3.5 at the genus level; columns represent differential metabolites. The top dendrogram represents the clustering of metabolites (columns), and the left dendrogram represents the clustering of bacterial genera (rows), both based on Euclidean distance and Ward’s linkage method, grouping highly correlated items together. Red = positive correlation, blue = negative correlation. * *p* < 0.05, *** *p* < 0.001.

Figure 8 shows the Spearman correlation heatmap for the selected differential bacterial genera and differential hepatic metabolites. The abundance of *Rikenella* was significantly and positively correlated with diisopropanolamine and N6,N6-dimethyladenosine (*p* < 0.05), and significantly and negatively correlated with allosecurinine, (Z)-2-octylpent-2-enedioic acid, glutathione, beta-alanyl-L-arginine, H-TYR-GLN-OH, Ser-Leu, H-Phe-Met-OH, glycylleucine, valylasparagine, carboxymethyl lysine, N-(3-amino-2-hydroxy-3-oxopropyl)-L-valine, serylmethionine, methionyl-glutamine, threonylmethionine, valylglutamine, Ile-Ser, dehydroeffusol, asparaginyl-phenylalanine, (S)-3-(3-(methylsulfonyl)phenyl)-1-propylpiperidine, L-leucyl-L-alanine, H-Val-Tyr-OH, glutaminylserine, valylserine, celgosivir, H-PHE-ASP-OH, flexibolide, Leu-Ala-Gly, N(5)-acetyl-L-ornithine, N-acetyl-L-aspartic acid, hydroxyacetone phosphate, acetylvalerenolic acid, alanylserine, 2-heptadecanone, arizonicanol A, and nubenolide acetate (*p* < 0.05).

For *Lactobacillus,* abundance was significantly positively correlated with methionine sulfoxide and His-Glu-Asn (*p* < 0.05), and significantly negatively correlated with serylmethionine, valylglutamine, Ile-Ser, H-PHE-ASP-OH and 2-O-methylascorbic acid (*p* < 0.05).

For *Bacteroides,* abundance was significantly positively correlated with diisopropanolamine and N6,N6-dimethyladenosine (*p* < 0.05), and significantly negatively correlated with 3-acetamino-6-isobutyl-2,5-dioxopiperazine, H-TYR-GLN-OH, Ser-Leu, H-Phe-Met-OH, glycylleucine, valylasparagine, carboxymethyl lysine, N-(3-amino-2-hydroxy-3-oxopropyl)-L-valine, serylmethionine, threonylmethionine, valylglutamine, Ile-Ser, dehydroeffusol, asparaginyl-phenylalanine, (S)-3-(3-(methylsulfonyl)phenyl)-1-propylpiperidine, L-leucyl-L-alanine, H-Val-Tyr-OH, glutaminylserine, valylserine, Leu-Ala-Gly, N(5)-acetyl-L-ornithine, 17beta-hydroxy-4-oxa-5alpha-estr-1-en-3-one acetate, Ile-Phe-Leu, 2-(acetylamino)-2-deoxy-6-O-sulfo-alpha-D-glucopyranose, and camphorsulfonic acid (*p* < 0.05).

For *Oscillibacter,* abundance was significantly negatively correlated with 3-acetamino-6-isobutyl-2,5-dioxopiperazine and H-TYR-GLN-OH (*p* < 0.05).

## 4. Discussion

Growth performance is the most direct indicator for evaluating poultry production, and the rearing system is a key non-genetic factor that can profoundly affect it [7]. Jin et al. [24] found that cage-reared Wannan Yellow chickens had significantly higher body weight, average daily feed intake (ADFI), and average daily gain (ADG), and a significantly lower feed conversion ratio (FCR), than free-range chickens. El-Deen et al. [25] reported that cage-reared broilers had significantly higher body weight, body weight gain, survival rate, and FCR, but no significant difference in feed intake, compared with free-range broilers [25]. El-Maaty et al. [26] showed that Cobb 500 broilers reared in cages exhibited significantly greater body weight and total weight gain than those reared on litter or plastic floor systems [26]. However, some studies have reported contradictory results. Yan et al. [27] found that during 1–36 days of age, Ross 308 broilers raised on floor litter or in plastic mesh cages showed higher ADG, ADFI, and FCR than those in multi-tier cages [27]. Dikmen et al. [20] reported that Ross 308 broilers reared on floor litter had significantly higher body weight and ADG, and a markedly lower feed-to-gain ratio, than those in slatted cages, with no significant difference in feed intake [20]. These discrepancies may be attributed to multiple factors, such as genotype, slaughter age, type of cage system, rearing method, litter material, stocking density, and indoor climatic conditions [20]. The results of the present study demonstrated that cage-reared broilers had significantly higher final body weight and ADG, and significantly lower ADFI and feed-to-gain ratio (F/G), compared with free-range broilers. This may be because free-range broilers are exposed to a variable environment, resulting in increased physical activity and higher energy expenditure, which in turn leads to a poorer feed conversion ratio and reduced fat deposition, ultimately causing lower body weight and ADG relative to cage-reared birds.

It is important to clarify that the reduced growth performance of free-range broilers should not be viewed merely as a negative outcome but as a distinctive physiological adaptive trait. Concurrent alterations in the cecal microbiota and hepatic metabolism provide critical insights into this adaptive mechanism. Specifically, the free-range environment may redirect metabolic resources away from rapid growth toward other higher-priority physiological processes, such as immune regulation or enhanced stress resistance, with these shifts manifesting as changes in microbial composition and hepatic metabolic pathways. Therefore, examining gut–liver axis function against the backdrop of diminished growth performance enables us to move beyond superficial phenotypic comparisons, thereby revealing, at a mechanistic level, how rearing environments shape host physiology.

Slaughter performance is a key indicator of meat production efficiency and economic value in poultry. Generally, broilers with good meat production performance should achieve a dressing percentage above 80% [28]. However, research findings on whether slaughter performance is affected by different rearing systems are inconsistent. Jin et al. [24] found that free-range Wannan Yellow chickens had significantly lower abdominal fat weight but higher breast and leg muscle yields than cage-reared birds. Sun et al. [5] reported that free-range Beijing-You chickens had higher breast and leg muscle weights and percentages, but lower abdominal fat weight and percentage, than cage-reared chickens. Xu et al. [21] showed that cage-reared Wenchang chickens exhibited significantly higher live weight, carcass weight, eviscerated weight, semi-eviscerated weight, breast muscle weight, abdominal fat weight, abdominal fat percentage, semi-eviscerated percentage, and eviscerated percentage than free-range birds. These results may be explained by the larger activity range of free-range chickens, where natural behaviors such as walking, running, and jumping can promote the development of breast and leg muscles, and increase overall physical activity and basal metabolic rate, leading to increased energy expenditure and reduced lipid accumulation. In addition to the rearing system, poultry slaughter performance is also influenced by breed, nutritional level, disease, and environment [29]. El-Maaty et al. [26] found no significant differences in slaughter performance among Cobb 500 broilers reared in cage, litter, and plastic floor systems. The results of the present experiment showed that the abdominal fat percentage was significantly decreased under the free-range system, while no significant differences were observed in the other slaughter performance indices between the two groups. The dressing percentage in both groups exceeded 80%, indicating that yellow-feathered broilers under both cage and free-range systems exhibited satisfactory meat production performance.

Serum biochemical parameters reflect the physiological and metabolic status of animals and are influenced by multiple factors, among which the rearing system is one of the most critical [30]. Ghanima et al. [31] reported that serum alanine aminotransferase (ALT) and aspartate aminotransferase (AST) levels were not significantly affected in broilers raised in cage, litter, or plastic slat systems. El-Maaty et al. [26] found no significant changes in any serum biochemical parameters in broilers raised in cage, litter, or plastic floor systems. The present study found no significant differences in any serum biochemical parameters between cage-reared and free-range broilers, consistent with the findings of Sogunle et al. [32]. This suggests that, under the conditions of this study, changes in the rearing system did not significantly alter broiler serum biochemical parameters, likely due to adaptive adjustments of the body to different rearing environments that maintain relatively stable levels of serum metabolites.

The gut microbiota plays a vital role in the animal intestinal tract and is closely associated with host physiological functions and health [33]. The rearing system can significantly influence the composition of the gut microbiota, thereby affecting the animal’s metabolic processes and ultimately leading to different health outcomes. When fed the same diet, free-range chickens are exposed to a more complex and diverse environment, including varied water sources and food, which alters their gut microecology [12]. Zhang et al. [4] investigated the effects of floor free-range and cage rearing on the gut microbiota of yellow-feathered broilers and found that *Firmicutes*, *Proteobacteria*, and *Bacteroidota* were the most common phyla in the intestinal tract under both rearing systems. Compared with caged broilers, floor free-range broilers showed greater gut microbial diversity, and the ratio of *Bacteroidota* to *Firmicutes*, as well as the abundances of *Helicobacter* and *Roseburia,* might influence broiler growth performance. The results of the present study indicated that the cecal microbial alpha diversity (Shannon and Simpson) was significantly higher in free-range yellow-feathered broilers than in the cage group, consistent with previous findings. However, beta diversity analysis revealed no significant separation in the overall microbial community structure between the two groups. This may be because the core microbiota remain relatively stable under different rearing systems. For example, the chicken gut microbiota is dominated by phyla such as *Firmicutes* and *Bacteroidota*, which are strongly influenced by host genetics and dietary composition [34]. In this study, broilers in both the free-range and cage groups were fed the same diet, which may be a key reason for the lack of significant beta diversity. The *Firmicutes/Bacteroidota* ratio in the free-range group was higher than that in the cage group, which would normally suggest greater fat deposition in free-range broilers [35]. However, measured abdominal fat deposition was lower in the free-range group than in the cage group. This discrepancy may be attributed to higher physical activity and increased energy expenditure under the free-range system, which reduced fat accumulation. In addition, broiler breed can also affect the *Firmicutes/Bacteroidota* ratio [36]. At the genus level, the free-range group was enriched with several potentially beneficial bacteria, including *Lactobacillus*, *Rikenella*, *Bacteroides*, and *Oscillibacter. Lactobacillus* can enhance intestinal barrier function and modulate host immunity [37]. *Rikenella* can strengthen tight junctions and intestinal barrier function [38,39] and may promote mineral absorption by lowering intestinal pH, thereby affecting bone metabolism [40]. *Bacteroides* is involved in polysaccharide degradation and short-chain fatty acid production [41]. *Alistipes* exerts protective effects against colitis, fibrosis, and other conditions [42]. An increased abundance of *Faecalibacterium* is a positive signal because it produces butyrate, maintains barrier integrity, and exhibits anti-inflammatory and immunomodulatory functions [43]. *Oscillibacter* can produce butyrate, which is essential for the energy supply of colonic epithelial cells [44]. Furthermore, *Barnesiella* can clear vancomycin-resistant enterococci from the intestinal tract, while *Megamonas* degrades inositol, promotes lipid absorption, and is associated with obesity [45]. In summary, the alterations in the cecal microbiota of yellow-feathered broilers at the genus level observed in this study, particularly the enrichment of butyrate-producing bacteria such as *Faecalibacterium*, indicate enhanced intestinal barrier function, a more stable microbial community structure, and an overall improvement in health status.

Hepatic metabolomics plays a vital role in animal nutrition and health assessment. Analyzing liver metabolites help clarify physiological status of animals under different environmental conditions, enabling the evaluation of their health and production performance. Pathway enrichment analysis revealed that the necroptosis pathway showed the most pronounced changes, accompanied by significant alterations in thiamine metabolism and in amino sugar and nucleotide sugar metabolism. In the necroptosis pathway, both sphingomyelin and sphingosine were significantly upregulated in the free-range group, a hallmark of disrupted sphingolipid metabolic homeostasis. Previous studies have shown that sphingomyelin accumulation can inhibit Akt signaling via protein phosphatase 2A (PP2A), thereby interfering with insulin sensitivity [46]. Disruption of the ceramide/sphingomyelin (Cer/SM) balance is associated with hepatocyte injury, dysregulated lipid metabolism, and insulin resistance [47]. However, because no significant differences in serum glucose or lipid parameters were observed between the two groups in this study, these mechanisms require further verification by examining the gene expression or enzymatic activity of ceramide synthase (CerS) and 3-mercaptopyruvate sulfurtransferase (3-MST) in the future.

In the amino sugar and nucleotide sugar metabolism pathway, UDP-L-arabinofuranose (UDP-Araf), D-glucosamine 6-phosphate, and galactose 1-phosphate (Gal-1-P) were all significantly upregulated. UDP-Araf has no known specific physiological function in animals [48]. D-Glucosamine 6-phosphate is a core intermediate of the hexosamine biosynthesis pathway; its upregulation indicates activation of this pathway, possibly in response to high nutrient stress, such as hyperglycemia or elevated free fatty acids [49]. Gal-1-P is a key intermediate in galactose metabolism. Excessive accumulation can inhibit UDP-glucose pyrophosphorylase (UGP), reducing levels of UDP-glucose and UDP-galactose, which are essential glycosyl donors for glycoprotein and glycolipid synthesis. Therefore, the significant upregulation of Gal-1-P suggests abnormal galactose metabolism, which may exacerbate overall metabolic stress [50].

In the thiamine metabolism pathway, thiamine monophosphate (TMP) and nicotinamide adenine dinucleotide (NAD) were both significantly upregulated. TMP is a phosphorylated derivative of vitamin B1 and can be converted to thiamine pyrophosphate (TPP) by thiamine monophosphate kinase (ThiL). TPP is a key coenzyme in carbohydrate and amino acid metabolism. Thus, the marked upregulation of TMP may imply impaired conversion to TPP, thereby affecting thiamine metabolism and related pathways [51]. NAD is an important regulator of cellular redox reactions and serves as a cofactor or co-substrate for various key enzymes [52]; its upregulation suggests enhanced cellular energy metabolism [53]. The concurrent alterations in TMP and NAD may reflect an interplay between suppression and compensatory activation of energy metabolism.

Correlation analysis showed that the abundances of enriched bacterial genera were significantly negatively correlated with multiple hepatic metabolites, and these correlated differential metabolites were mostly intermediates or end products of amino acid metabolism. This negative correlation pattern aligns with the PICRUSt-predicted nutrient scavenging function of the microbiota in the free-range group, indicating that the microbial community in free-range chickens is functionally adapted to nutrient recycling. Specifically, these bacteria may use amino acids or nitrogenous compounds as energy or nitrogen sources, thereby reducing the abundance of amino acid-derived metabolites in the liver [22,54]. Such microbial activities may contribute to the alterations in amino acid metabolism and energy homeostasis observed in free-range broilers.

## 5. Conclusions

Free-range rearing reduced growth performance and abdominal fat deposition in yellow-feathered broilers but increased cecal microbial diversity and improved hepatic nutrient metabolism. These results suggest that environmental changes alter both the cecal microbiota and hepatic metabolism in yellow-feathered broilers, and that these two components may jointly regulate host metabolic flexibility and environmental adaptability.

## Figures and Tables

**Table 1:** Composition and nutrient levels of the basal diet (air-dry basis).

Items	Grower (22–42 Days)	Finisher (43–63 Days)
Ingredients (%)		
Corn	58.17	58.93
Wheat bran	2.80	3.00
Cottonseed meal	14.70	11.90
Corn protein meal	5.00	5.00
Wheat flour	10.00	10.00
Corn germ meal	3.60	4.80
CaHPO_4_	1.02	0.75
Limestone	1.26	1.12
Cottonseed oil	1.60	2.80
NaCl	0.20	0.20
Lysine	0.63	0.63
Methionine	0.26	0.16
Threonine	0.1	0.08
Premix ^1^	0.66	0.63
Total	100.00	100.00
Nutrient levels ^2^		
ME (MJ/kg)	12.54	12.96
CP (%)	17.85	16.23
Ca (%)	1.25	1.00
TP (%)	0.84	0.76
Lysine (%)	1.07	0.91
Methionine (%)	0.50	0.46
Met + Cys (%)	0.86	0.84

^1^ The premix provided the following nutrients per kilogram of diet: vitamin A 12,000 IU, vitamin D_3_ 2500 IU, vitamin E 15 IU, vitamin K_3_ 2.2 mg, vitamin B_1_ 2.2 mg, vitamin B_2_ 5.5 mg, vitamin B_12_ 0.02 mg, niacin 35 mg, calcium pantothenate 12 mg, folic acid 1.20 mg, biotin 0.15 mg, choline 1200 mg, Fe 80 mg, Mn 78 mg, Zn 72 mg, Cu 8 mg, Se 0.20 mg, I 0.40 mg. ^2^ Crude protein was measured, whereas all other nutrient values were calculated.

**Table 2:** Effects of different rearing systems on growth performance of yellow-feathered broilers.

Items ^1^	LY	SY	*p*-Value
22 d WG, g	617.81 ± 20.07	619.71 ± 1.98	0.798
63 d WG, g	3112.29 ± 39.72 ^b^	3016.67 ± 73.06 ^a^	0.006
ADFI, g	134.32 ± 4.28 ^a^	139.01 ± 2.70 ^b^	0.020
ADG, g	59.39 ± 0.88 ^b^	57.07 ± 1.74 ^a^	0.005
F/G	2.26 ± 0.07 ^a^	2.44 ± 0.08 ^b^	<0.001

^a, b^ Means with different superscripts with in the same row differ significantly (*p* < 0.05). ^1^ WG: fasted body weight; ADG: average daily gain; ADFI: average daily feed intake; F/G: feed to gain ratio. LY = cage group; SY = free-range group.

**Table 3:** Effects of different rearing systems on serum biochemical parameters of yellow-feathered broilers.

Items ^1^	LY	SY	*p*-Value
TP, g/L	37.97 ± 2.61	37.57 ± 5.83	0.919
ALB, g/L	14.30 ± 1.18	14.23 ± 2.31	0.967
GLB, g/L	23.67 ± 1.43	23.33 ± 3.67	0.891
BUN, mmol/L	0.40 ± 0.03	0.35 ± 0.05	0.177
TG, mmol/L	1.39 ± 0.18	1.21 ± 0.03	0.166
T-CHO, mmol/L	3.66 ± 0.47	4.20 ± 0.90	0.406
HDL-c, mmol/L	2.37 ± 0.54	2.69 ± 0.38	0.453
LDL-c, mmol/L	1.00 ± 0.29	1.15 ± 0.85	0.782
ALT, U/L	3.33 ± 0.58	4.00 ± 1.00	0.374
AST, U/L	260.67 ± 32.33	265.67 ± 40.70	0.876
GLU, mmol/L	14.22 ± 0.53	14.29 ± 1.34	0.937

^1^ TP: total protein; ALB: albumin; GLB: globulin; BUN: urea nitrogen; TG: triglycerides; T-CHO: total cholesterol; HDL-c: high-density lipoprotein cholesterol; LDL-c: low-density lipoprotein cholesterol; ALT: alanine aminotransferase; AST: aspartate aminotransferase; GLU: glucose. LY = cage group; SY = free-range group.

**Table 4:** Effects of different rearing systems on slaughter performance of yellow-feathered broilers.

Items ^1^	LY	SY	*p*-Value
Dressing percentage, %	91.94 ± 1.96	91.15 ± 1.08	0.283
Semi-eviscerated percentage, %	82.66 ± 1.70	82.16 ± 1.51	0.499
Eviscerated percentage, %	69.54 ± 2.10	68.52 ± 2.28	0.309
Breast muscle percentage, %	17.27 ± 2.14	16.22 ± 2.09	0.281
Leg muscle percentage, %	19.81 ± 1.41	19.65 ± 1.14	0.777
Abdominal fat percentage, %	4.48 ± 1.21 ^b^	3.50 ± 0.39 ^a^	0.026

^a, b^ Means with different superscripts with in the same row differ significantly (*p* < 0.05). ^1^ LY = cage group; SY = free-range group.

## Data Availability

The datasets of the current study and the models used are available from the corresponding author upon reasonable request.

## Supplementary Materials

The following supporting information can be downloaded at: https://www.mdpi.com/article/10.3390/ani16121920/s1, Table S1: Comparison of the relative abundance of dominant bacterial phyla in the cecal microbiota of yellow-feathered broilers under different rearing systems; Table S2: Comparison of the relative abundance of dominant bacterial genera in the cecal microbiota of yellow-feathered broilers under different rearing systems; Table S3: Differential metabolites in the positive ion mode; Table S4: Differential metabolites in the negative ion mode.
