# Supplementary material for: Effects of Different Rearing Systems (Cage vs. Free-Range) on Growth Performance, Serum Biochemical Parameters, Slaughter Performance, Cecal Microbiota, and Hepatic Metabolism of Yellow-Feathered Broilers
Source: Animals (Basel). 2026 Jun 21;16(12):1920. doi: 10.3390/ani16121920 (PMC13295336; doi:10.3390/ani16121920)
Supplement: Supplementary file 1 [file animals-16-01920-s001.zip › animals-4365693-supplementary.pdf]

Table S1. Comparison of the relative abundance of dominant bacterial phyla in the cecal microbiota of yellow-feathered broilers under different rearing systems.

Items [1]	LY	SY	*p*-Value
Bacteroidota	56.419±14.981	46.678±4.383	0.200
Firmicutes	41.048±14.880	51.181±4.007	0.180
Desulfobacterota	1.414±1.546	1.100±1.055	0.717
Actinobacteriota	0.548±0.604	0.235±0.136	0.291
Proteobacteria	0.429±0.269	0.481±0.261	0.765
Cyanobacteria	0.013±0.013[a]	0.277±0.254[b]	0.049
Campylobacterota	0.122±0.222	0.043±0.083	0.476

[a, b] Means with different superscripts with in the same row differ significantly ($p < 0.05$). [1] LY=cage group; SY= free-range group.

Table S2. Comparison of the relative abundance of dominant bacterial genera in the cecal microbiota of yellow-feathered broilers under different rearing systems.

Items [1]	LY	SY	*p*-Value
Alistipes	37.059±17.743	18.587±5.852	0.058
Barnesiella	12.119±20.784	9.866±9.469	0.831
Bacteroides	5.065±3.512	12.927±7.379	0.064
Megamonas	8.998±11.286	3.040±6.611	0.338
Faecalibacterium	3.726±2.331	6.373±5.745	0.368
Ligilactobacillus	1.007±0.988	1.285±2.111	0.797
Lactobacillus	0.282±0.192[a]	2.572±1.849[b]	0.025
Rikenella	0.416±0.389[a]	2.120±1.566[b]	0.046
Bilophila	1.385±1.542	1.065±1.034	0.710
[Ruminococcus]_torques_group	1.820±1.325	1.505±1.041	0.687

[a, b] Means with different superscripts with in the same row differ significantly ($p < 0.05$). [1] LY=cage group; SY= free-range group.

Table S3. Differential metabolites in the positive ion mode.

Differential metabolites	VIP	Fold change	*p*-value	Change trend
L-xylo-hex-3-ulono-1,4-lactone	1.991	32.149	<0.001	up
Threonylmethionine	1.918	9.133	<0.001	up
Loline	1.907	1.710	<0.001	up
3-Glucosyl-2,3',4,4',6-pentahydroxybenzophenone	1.909	1.683	<0.001	up
N-(3-Benzooxazol-2-yl-4-hydroxy-phenyl)-2-p-tolyloxyacetamide	1.925	1.835	<0.001	up
Aspartylglycosamine	1.887	2.090	<0.001	up
Sulcatin	1.921	0.649	<0.001	down
H-Val-Met-OH	1.903	2.662	<0.001	up
DL-Leucyl-DL-phenylalanine	1.867	2.567	<0.001	up
Leptosin S	1.839	0.249	<0.001	down
3,7-Dimethylguanine	1.853	0.316	<0.001	down
H-Phe-Met-OH	1.843	2.308	<0.001	up
Serylmethionine	1.846	5.230	<0.001	up
Fluoroquinolone carboxylic	1.841	1.597	<0.001	up
8-MeSO-octyl-CO2H	1.823	0.438	<0.001	down
5-hydroxyfuran-2-carboxylic Acid	1.811	1.956	<0.001	up
Carboxy methyl lysine	1.811	3.564	0.001	up
Methionyl-Glutamine	1.827	4.801	0.001	up
Lactaroviolin	1.810	1.872	0.001	up
Ro 20-1724	1.809	2.444	0.001	up
N-Acetyl-L-histidine	1.792	1.722	0.001	up
Harmane	1.800	2.271	0.001	up
Valylglutamine	1.788	3.453	0.001	up
L-Valyl-L-phenylalanine	1.792	2.338	0.001	up

Methionyl-Asparagine	1.785	3.404	0.001	up
(+/-)-Dihydroactinidiolide	1.781	0.365	0.001	down
Esprocarb	1.768	2.313	0.001	up
Valylthreonine	1.751	3.889	0.001	up
Benzylurea	1.778	2.605	0.001	up
Ile Ile	1.749	1.843	0.001	up
(-)-Salsolinol	1.801	4.301	0.001	up
Phe-Ala-Gly	1.735	2.156	0.002	up
3-Mercaptopyruvic acid	1.735	1.545	0.002	up
5'-Demethoxycadensin G	1.748	0.570	0.002	down
Glutathione	1.718	2.259	0.002	up
H-MET-TRP-OH	1.729	2.358	0.002	up
Thiomorpholine 3-carboxylate	1.712	0.219	0.002	down
Glycylleucine	1.749	1.805	0.002	up
Leu-Thr	1.762	2.992	0.002	up
Ile-Ser	1.756	5.276	0.002	up
Glycyl-Methionine	1.740	2.610	0.002	up
N-(3-Amino-2-hydroxy-3-oxopropyl)-L-valine	1.745	4.633	0.002	up
Leuhistin	1.693	2.028	0.003	up
6-Hydroxyazapropazone	1.688	0.277	0.003	down
D-Lysopine	1.742	2.670	0.003	up
Minoxidil	1.715	3.659	0.003	up
Fluzinamide	1.684	3.183	0.003	up
Leucylleucine methyl ester	1.721	1.721	0.003	up
Serylvaline	1.730	2.320	0.003	up
Metiamide	1.695	2.011	0.003	up

N-acetylleucine	1.680	0.590	0.003	down
all-trans-4-Oxoretinoic acid	1.720	1.621	0.003	up
Thr Leu	1.683	2.747	0.004	up
Phe Thr	1.696	2.458	0.004	up
Phenylalanylvaline	1.673	2.133	0.004	up
chaetoglocin D	1.752	0.566	0.004	down
Diisopropanolamine	1.667	0.332	0.004	down
(-)-7-Hydroxy-beta-isosparteine	1.703	1.527	0.004	up
Arabinosylhypoxanthine	1.675	2.542	0.004	up
H-Thr-Phe-OH	1.646	2.175	0.004	up
3-methylglutaconate	1.647	0.661	0.004	down
Quetiapine	1.759	1.545	0.005	up
3-Sulfopyruvic acid	1.648	2.221	0.005	up
Ser Leu	1.647	2.582	0.005	up
N6,N6-Dimethyladenosine	1.647	0.567	0.005	down
Tryptophyl-Cysteine	1.651	2.286	0.005	up
20-Dihydrodydrogesterone	1.624	0.245	0.005	down
3-[4-(3-Trifluoromethyl-phenyl)-piperazin-1-yl]-dihydrofuran-2-one	1.621	1.581	0.005	up
2-Methyl-3H-quinazolin-4-one	1.650	1.903	0.005	up
Montiporyne F	1.621	0.270	0.006	down
Dipeptide 2	1.628	2.288	0.006	up
deoxycarnitine	1.720	1.635	0.006	up
H-Ile-Tyr-OH	1.656	1.906	0.006	up
Apo-13-zeaxanthinone	1.599	0.619	0.007	down
H-Leu-Asn-OH	1.680	1.838	0.007	up
5-methyluridine	1.664	1.578	0.007	up

Serylserine	1.593	2.057	0.007	up
3-Hydroxy-4-hydroxymethyl-2-methylpyridine-5-carboxylate	1.603	1.525	0.007	up
Salidroside	1.678	4.610	0.008	up
3-Hydroxyhexobarbital	1.601	2.124	0.008	up
Adenosine-2'-monophosphate	1.623	0.346	0.008	down
Dihydrocoumarin	1.609	1.656	0.008	up
(Z)-2-octylpent-2-enedioic acid	1.583	1.659	0.008	up
N-Nitrosoguvacoline	1.578	1.501	0.008	up
N-Linoleoyl Glutamic acid	1.576	0.296	0.009	down
H-TYR-GLN-OH	1.628	1.528	0.009	up
gamma-Glutamyl-S-methylcysteine	1.644	6.762	0.009	up
Cysteinylglycine	1.562	2.106	0.010	up
4-Hydroxyproline	1.634	1.565	0.010	up
2-Amino-3-methyl-1-pyrrolidin-1-YL-butan-1-one	1.691	3.199	0.010	up
Phenylalanyltryptophan	1.567	1.887	0.010	up
Serinyl-Gamma-glutamate	1.552	1.718	0.010	up
Prolyl-tyrosine	1.599	0.652	0.010	down
13alpha-Angeloyloxylupanine	1.547	6.338	0.011	up
D-Ala-D-Ala	1.554	2.096	0.011	up
Anacine	1.542	0.309	0.011	down
2,4-Thiazolidinedicarboxylic acid, 2-methyl-	1.569	0.069	0.011	down
Polyporusterone B	1.590	4.524	0.012	up
allo-Protolichesterinic acid	1.578	0.171	0.012	down
Hypusine	1.528	0.612	0.013	down
S-(Formylmethyl)glutathione	1.606	2.253	0.013	up
D,L-Buthionine	1.589	2.445	0.013	up

3-Mercapto-2-methylpentanal	1.582	1.604	0.013	up
methionine sulfoxide	1.520	0.472	0.013	down
1-(2-Thienyl)-1-heptanone	1.587	2.121	0.014	up
Ala-Val	1.529	2.225	0.014	up
6-Hydroxy-3,4-dihydro-1-oxo-beta-carboline	1.582	0.543	0.014	down
Tyr Met	1.568	1.973	0.014	up
N2,N5-Dibenzoyl-L-ornithine	1.534	1.538	0.014	up
H-ILE-TRP-OH	1.552	1.854	0.014	up
Aminofurantoin	1.519	0.324	0.015	down
Hypotaurine	1.584	1.757	0.015	up
3-acetamino-6-isobutyl-2,5-dioxopiperazine	1.498	1.634	0.015	up
Prolylproline	1.500	0.520	0.015	down
Threonylglutamine	1.510	1.957	0.015	up
2-(3-Methylbut-2-enyl)-4-(2-methylbut-3-en-2-yl)phenol	1.533	0.426	0.016	down
Crystal violet	1.488	0.335	0.016	down
Suspensoside B	1.476	0.304	0.017	down
(2E)-3-Methylpent-2-enedioylcarnitine	1.513	0.238	0.017	down
S-Hydroxymethylglutathione	1.569	2.012	0.018	up
Hypophyllanthin	1.479	2.310	0.018	up
DL-alpha,epsilon-Diaminopimelic acid	1.526	2.153	0.018	up
2-Dehydrococcinelline	1.621	1.513	0.018	up
Propofol glucuronide	1.483	0.362	0.018	down
H-beta-Ala-Tyr-OH	1.571	2.040	0.018	up
alpha-Hydrojuglone-4-glucoside	1.530	0.461	0.018	down
Dehydrofalcarinol	1.473	0.363	0.018	down
cis-4,7,10,13,16-Docosapentaenoic acid	1.466	0.441	0.018	down

Prenisteine	1.477	1.828	0.018	up
Acanthovagasteroid A	1.513	2.290	0.019	up
Valylasparagine	1.542	4.092	0.019	up
3-(2,4-Cyclopentadien-1-ylidene)-5alpha-androstan-17beta-ol	1.495	0.462	0.019	down
Stoloniferone G	1.456	0.285	0.019	down
1-Octanethiol	1.574	1.564	0.020	up
Chloropyramine	1.452	0.383	0.021	down
6alpha-Hydroxyundulatine	1.454	1.617	0.022	up
Glycyrrhizaisoflavone A	1.492	0.392	0.022	down
Linalyl acetate	1.566	1.778	0.023	up
Sabinic acid	1.481	1.878	0.023	up
L-Alanyl-L-leucine	1.439	1.679	0.024	up
(9Z,12Z)-3-Hydroxyhexadecadienoylcarnitine	1.455	0.225	0.024	down
Nicotinamide adenine dinucleotide (NAD)	1.498	2.572	0.025	up
Ramiflorine A	1.458	0.384	0.025	down
Thiamine monophosphate	1.489	2.342	0.025	up
H-Ser-Tyr-OH	1.409	3.073	0.025	up
2-Methyl-2-[(1-oxo-2-propenyl)amino]-1-propanesulfonic acid	1.433	0.389	0.026	down
N(4)-acetyl-2'-deoxycytidine	1.433	1.528	0.026	up
Tritoniopsin D	1.431	0.413	0.026	down
Tricalysioside T	1.427	0.408	0.027	down
Lys Tyr Phe	1.401	0.544	0.028	down
Germacrone	1.423	0.412	0.028	down
Helogenin	1.401	0.259	0.029	down
H-Pro-Trp-OH	1.468	0.511	0.029	down
Taurodeoxycholate	1.420	0.412	0.029	down

Eicosapentaenoic acid ethyl ester	1.390	0.442	0.030	down
1,3-DIMETHYLURIC ACID	1.412	1.686	0.030	up
Arachidonoyl-carnitine	1.456	0.356	0.030	down
28-Homobrassinolide	1.383	0.246	0.030	down
beta-Alanyl-L-arginine	1.386	2.126	0.031	up
Clopidogrel	1.468	0.575	0.031	down
alpha,alpha'-Diethyl-4,4'-bis(2-propynyloxy)stilbene	1.393	0.525	0.032	down
Threonyltyrosine	1.378	3.853	0.032	up
(9Z)-3-hydroxydodecenoylcarnitine	1.377	0.214	0.033	down
Yakuchinone A	1.483	1.636	0.033	up
7,4'-Di-O-methylapigenin	1.417	1.700	0.034	up
(8E,15E)-1,8,15-Heptadecatriene-11,13-diyne	1.374	0.393	0.034	down
Adenosine	1.407	1.514	0.034	up
Threonylserine	1.360	1.571	0.035	up
(7Z,9E)-Dodeca-7,9-dienoylcarnitine	1.353	0.097	0.035	down
O-fumaryl-L-carnitine	1.462	1.564	0.035	up
cis-4,7,10,13,16,19-Docosahexaenoic acid	1.346	0.442	0.036	down
Hirsutalin E	1.347	0.650	0.037	down
Lanthionine ketimine	1.386	3.334	0.038	up
Threonylthreonine	1.372	1.737	0.038	up
1-(beta-D-Ribofuranosyl)-1,4-dihydronicotinamide	1.406	0.123	0.038	down
SM(d18:0/18:1(9Z))	1.338	1.904	0.039	up
sn-Glycero-3-phosphocholine	1.419	1.520	0.040	up
Anthracene-9-carboxylic acid	1.343	0.595	0.040	down
Mupirocin	1.358	0.549	0.040	down
Stephalonine A	1.330	4.022	0.040	up

2,6-dimethoxybenzoylsalicylhydrazide	1.338	0.664	0.040	down
sphingosine	1.323	1.522	0.040	up
His Glu Asn	1.418	0.334	0.041	down
Broussonetine M1	1.326	0.536	0.042	down
Pierisformoside A	1.345	0.258	0.043	down
Aglacin K	1.357	0.603	0.043	down
(6R,9S)-3-Oxo-alpha-ionol beta-D-glucoside	1.326	0.284	0.043	down
MG(0:0/24:6(6Z,9Z,12Z,15Z,18Z,21Z)/0:0)	1.320	1.832	0.044	up
Pterosin B	1.387	0.537	0.044	down
PE(16:0/18:2(9Z,12Z))	1.413	1.502	0.045	up
DPPC	1.320	1.643	0.045	up
Lumichrome	1.320	0.648	0.045	down
Lys Val Phe	1.312	0.273	0.045	down
25-Hydroxyvitamin D3 3-sulfate ester	1.347	0.265	0.045	down
Prostaglandin E1	1.316	0.622	0.045	down
N-Acetylserine	1.352	0.640	0.046	down
Catechol glucuronide	1.396	0.305	0.047	down
isobutyrylcarnitine (C4)	1.325	0.408	0.047	down
Allosecurinine	1.413	2.209	0.047	up
Asparaginyl-Valine	1.417	1.974	0.047	up
2,5-diaminohexanoic acid	1.293	1.718	0.047	up
L-Alanyl-L-tryptophan	1.306	1.695	0.048	up
Propionic acid, 2-methyl-2-((piperidinomethyl)thio)-	1.287	1.726	0.048	up
5-Methoxytryptophol	1.368	0.604	0.049	down
Spermine	1.302	0.539	0.049	down
H-Tyr-Phe-OH	1.339	1.756	0.049	up

O-Arachidonoyl Ethanolamine	1.290	0.526	0.049	down
Enalaprilat	1.341	0.518	0.049	down
LysoPE(0:0/22:5(7Z,10Z,13Z,16Z,19Z))	1.295	0.563	0.050	down

Table S4. Differential metabolites in the negative ion mode.

Differential metabolites	VIP	Fold change	*p*-value	Change trend
N-[1.3-dihydroxyoctadec-4-en-2-yl]octadecanamide	1.732	2.345	<0.001	up
L-Ascorbic acid	1.778	107.638	<0.001	up
Irisoquin F	1.684	5.241	<0.001	up
3-Vinyl-4H-1,2-dithiin	1.681	1.807	<0.001	up
Threonyltryptophan	1.754	3.405	<0.001	up
Pyridoxal phosphate	1.661	0.261	<0.001	down
Dehydroeffusol	1.605	3.695	<0.001	up
Acetylenedicarboxylate	1.663	1.638	<0.001	up
Fluorescein	1.696	2.450	<0.001	up
Asparaginyl-Methionine	1.592	3.193	<0.001	up
Lawinal	1.614	2.148	<0.001	up

Ser Phe	1.654	3.243	<0.001	up
H-Val-Val-OH	1.578	2.949	<0.001	up
H-LEU-ILE-OH	1.565	2.963	<0.001	up
Irisoquin E	1.596	2.100	<0.001	up
SM(d16:1/17:0)	1.618	2.738	<0.001	up
H-Ile-Asn-OH	1.595	2.451	<0.001	up
Parabanic acid	1.549	1.610	0.001	up
L-Leucyl-L-Valine	1.573	3.018	0.001	up
Fenthion oxon	1.647	2.676	0.001	up
Viridamine	1.592	1.545	0.001	up
PE-NMe2(16:0/18:2(9Z,12Z))	1.551	2.501	0.001	up
SM(d19:1/16:0)	1.552	2.602	0.001	up
L-Leucyl-L-alanine	1.541	2.976	0.001	up
Asparaginyl-Phenylalanine	1.529	3.359	0.001	up
Tryptophyl-Isoleucine	1.575	2.567	0.001	up
C16-Ceramide	1.505	1.871	0.001	up
2-Propanoylthiophene	1.541	1.733	0.001	up

Colupulone	1.602	5.629	0.001	up
Glutaminylserine	1.487	2.638	0.002	up
N-Acetylasparagine	1.521	1.769	0.002	up
N-Nitrosodi-n-butylamine	1.576	3.162	0.002	up
PE-NMe2(16:0/16:0)	1.489	2.269	0.002	up
H-Val-Tyr-OH	1.481	2.090	0.002	up
4-(Aminomethyl)-1-methylpiperidin-4-ol	1.506	3.441	0.002	up
gamma-Glu-Ile	1.475	2.228	0.002	up
Phenylalanylalanine	1.586	2.807	0.002	up
Leu-Ala-Gly	1.520	3.394	0.002	up
Enaminomycin A	1.473	2.337	0.002	up
Isopongaflavone	1.478	2.274	0.002	up
Seryltryptophan	1.518	2.303	0.002	up
N-Nitrosoproline	1.499	2.459	0.002	up
(3beta,22E,24R)-3-Hydroxyergosta-5,8,22-trien-7-one	1.564	1.521	0.002	up
Magnaldehyde D	1.513	2.322	0.002	up
Picrocrocin	1.494	1.881	0.002	up

Clonostachydiol	1.464	1.868	0.002	up
NALPHA-ACETYL-L-LYSINE	1.493	2.822	0.002	up
4-Nitrophenol	1.497	1.689	0.003	up
Pioglitazone	1.464	1.584	0.003	up
Salicylaldehyde	1.455	1.504	0.003	up
Polyporusterone G	1.496	2.033	0.003	up
Glutaminylglutamine	1.459	2.995	0.003	up
Tyr Leu	1.475	2.351	0.003	up
1,2-Cyclohexanedione	1.500	1.658	0.003	up
Menadione bisulfite	1.527	2.507	0.003	up
2-Hydroxy-3-methylbutyric acid	1.478	1.658	0.003	up
2-hydroxytridecanoic acid	1.472	1.782	0.003	up
PE(P-16:0/20:4(5Z,8Z,11Z,14Z))	1.519	2.769	0.003	up
Dihydroconiferin	1.507	2.004	0.003	up
4-Nitrocatechol	1.653	2.612	0.003	up
Glutaminylthreonine	1.430	2.672	0.004	up
Eurycolactone E	1.479	4.966	0.004	up

Perfluorobutanesulfonic acid	1.433	1.542	0.004	up
Caiophoraenin	1.421	1.725	0.004	up
Buturon	1.473	1.846	0.004	up
CDP-ethanolamine	1.419	2.470	0.004	up
3-Hydroxysebacic acid	1.437	1.568	0.005	up
Phenylalanyllysine	1.434	1.730	0.005	up
H-Pro-Asp-OH	1.451	7.475	0.005	up
Valylserine	1.422	3.342	0.005	up
Acetylvalerenolic acid	1.392	3.406	0.005	up
N-Desmethylaminopyrine	1.486	2.426	0.005	up
cis-7,10,13,16,19-Docosapentaenoic acid	1.388	4.055	0.005	up
CPA(18:0/0:0)	1.421	2.146	0.006	up
3-O-phosphonohexaric acid	1.447	1.783	0.006	up
Pierisformosin B	1.385	2.072	0.006	up
N(5)-Acetyl-L-ornithine	1.446	2.469	0.006	up
Dendronpholide N	1.414	2.108	0.006	up
5-epi-Valiolol 7-phosphate	1.487	1.871	0.006	up

D-Glucosamine 6-phosphate	1.406	1.794	0.006	up
Phe Gly Ile	1.389	1.844	0.006	up
(+)-Neomethynolide	1.429	1.911	0.006	up
Cepharadione B	1.442	5.177	0.006	up
(S)-3-(3-(Methylsulfonyl)phenyl)-1-propylpiperidine	1.425	2.538	0.006	up
Isocitrate	1.476	1.800	0.006	up
5-Hydroxymethylcytidine	1.448	2.803	0.006	up
Cinnzeylanol	1.410	1.557	0.006	up
Celgosivir	1.403	1.858	0.007	up
N-lactoyl-Methionine	1.378	0.557	0.007	down
Gymconopin D	1.467	2.184	0.007	up
Hippuric acid	1.378	2.033	0.007	up
Annuionone B	1.374	1.789	0.007	up
Hippeastrine	1.404	2.781	0.007	up
2,6-Di-tert-butyl-4-nitrophenol	1.429	1.606	0.007	up
Valylaspartic acid	1.401	3.521	0.007	up
N-acetyldopamine	1.367	3.799	0.007	up

beta-dibutyrin	1.418	1.671	0.007	up
Loureirin A	1.364	1.969	0.008	up
2-Hydroxydocosanoic acid	1.471	1.976	0.008	up
Cellulose, microcrystalline	1.428	1.874	0.008	up
3-(3,4-Dihydroxyphenyl)-1-propanol 3'-glucoside	1.377	2.516	0.008	up
Angulifolin C	1.364	1.999	0.008	up
Monoisodecyl phthalate	1.417	7.548	0.009	up
Kobusone	1.420	1.711	0.009	up
Aspartyl-Gamma-glutamate	1.364	1.784	0.009	up
Flurbiprofen	1.414	1.969	0.009	up
Arbutin	1.348	2.149	0.009	up
Isomatteucinol	1.391	2.233	0.009	up
(S)-Laudanosine	1.357	1.821	0.009	up
Cordobic acid 18-acetate	1.397	2.158	0.009	up
Spongianolide B	1.346	2.903	0.009	up
Pharboside D	1.371	1.899	0.009	up
Nortricycloekasantalic acid	1.346	1.734	0.009	up

Glycyl-L-tyrosine	1.405	1.869	0.009	up
CPA(16:0/0:0)	1.484	1.842	0.010	up
6-O-Acetylaustroinulin	1.369	0.580	0.010	down
1-Tert-butyl-3-(4-chlorophenyl)-1H-pyrazolo[3,4-d]pyrimidin-4-amine	1.442	2.332	0.010	up
NAGABA	1.458	0.358	0.010	down
Eterobarb	1.431	2.019	0.010	up
Methynolide	1.357	2.169	0.010	up
Leucyl-Histidine	1.339	1.753	0.010	up
LysoPE(P-18:1(9Z)/0:0)	1.548	1.909	0.010	up
Phe-Trp-Ala	1.332	1.538	0.010	up
Isobutylparaben	1.402	1.553	0.010	up
Buxifoliadine-D	1.400	2.066	0.010	up
3-Hydroxyphenylacetate	1.326	1.554	0.010	up
Alstonoxine B	1.325	1.815	0.011	up
Hexylresorcinol	1.382	1.638	0.011	up
Musk ambrette	1.369	2.195	0.011	up
Ribose-1-arsenate	1.338	2.161	0.011	up

Ancistrolikokine D	1.385	1.678	0.011	up
Penifulvin D	1.324	1.562	0.011	up
UDP-L-arabinofuranose	1.330	2.953	0.011	up
N-(1,3-dihydroxyoctadec-4-en-2-yl)tetradecanamide	1.378	2.460	0.011	up
Hydroxyacetone phosphate	1.380	1.785	0.011	up
N-Acetyl-L-glutamic acid	1.322	1.621	0.011	up
2-Deoxy-D-glucopyranose 6-phosphate	1.313	1.555	0.011	up
Metazocine	1.407	2.197	0.011	up
PE(18:1(9Z)/16:0)	1.326	1.770	0.011	up
N-Acetyl-L-aspartic acid	1.311	2.313	0.011	up
Oleyl sarcosine	1.422	0.411	0.012	down
D-Arabinose-5-phosphate	1.332	2.340	0.012	up
Pestalotiopamide E	1.328	1.506	0.012	up
Curvularin	1.403	2.316	0.012	up
Perindopril Acyl-beta-D-glucuronide	1.311	2.500	0.012	up
Apomine	1.316	0.480	0.012	down
LysoPC(0:0/18:0)	1.610	1.858	0.012	up

5-Chloro-2'-deoxyuridine	1.352	2.287	0.012	up
Huperzine S	1.380	1.625	0.012	up
N-Acetyldehydroanonaine	1.361	1.747	0.012	up
H-LEU-LEU-GLY-OH	1.321	1.841	0.012	up
Dimethyl dimethoxy biphenyl	1.313	1.972	0.012	up
(4-tert-Butyl-phenoxy)-acetic acid	1.385	1.566	0.012	up
Cotinine N-oxide	1.371	2.065	0.013	up
2-(Acetylamino)-2-Deoxy-6-O-Sulfo-alpha-D-Glucopyranose	1.317	1.758	0.013	up
Phorbol	1.327	2.216	0.013	up
Glutaminyltyrosine	1.327	1.721	0.013	up
Perfluorooctanoic acid	1.321	1.863	0.013	up
Methyl Eugenol	1.385	1.929	0.013	up
Desacetyltetraneurin D 15-O-isobutyrate	1.338	3.172	0.014	up
Alanylserine	1.291	1.887	0.014	up
N-glycoloyl-D-glucosamine	1.302	1.744	0.014	up
Norcyclocitrinol A	1.321	3.341	0.014	up
Cedpht	1.291	1.932	0.014	up

7-methoxy-6-(1,2,3-trihydroxy-3-methylbutyl)chromen-2-one	1.395	3.267	0.014	up
1,2,3,4-Tetrahydro-b-carboline-1,3-dicarboxylic acid	1.298	1.701	0.015	up
(x)-2-Heptanol glucoside	1.283	2.418	0.015	up
N,N'-Diacetylbenzidine	1.330	3.665	0.015	up
Hydrastinine	1.392	1.982	0.015	up
Alternapyrone B	1.358	2.967	0.015	up
Dinor-PGF2alpha	1.318	1.695	0.015	up
N-Linoleoyl Leucine	1.288	0.551	0.016	down
Kadcoccilactone E	1.320	3.019	0.016	up
Alfadolone	1.337	4.638	0.016	up
Nigerapyrone C	1.293	1.505	0.016	up
Arizonicanol A	1.289	2.543	0.016	up
Glutamylasparagine	1.365	2.884	0.017	up
Monoketocholic acid	1.327	1.863	0.017	up
2-O-Methylascorbic acid	1.544	1.944	0.017	up
(+)-Ambruticin	1.280	2.167	0.017	up
Ouabain	1.308	0.412	0.017	down

Ranunculin	1.265	2.688	0.017	up
7-Hydroxy-10-ethoxydehydrodihydrobotrydial	1.360	3.031	0.018	up
3-hydroxypentadecanoic acid	1.303	1.531	0.018	up
N-Feruloylserotonin	1.309	3.476	0.018	up
Phenylalanylhistidine	1.298	1.888	0.018	up
Perfluorobutanoic acid	1.261	1.876	0.018	up
Semiplenamide E	1.292	0.451	0.018	down
(+)-Neomenthyl O-beta-D-glucoside	1.284	1.786	0.019	up
H-PHE-ASP-OH	1.260	2.382	0.019	up
Glycyl-L-phenylalanine	1.259	2.132	0.019	up
P-Toluenesulfonic acid	1.281	1.530	0.020	up
8-Ethoxysachaconitine	1.245	0.478	0.020	down
Bruguierol C	1.361	2.587	0.020	up
Oxyphencyclimine	1.267	0.232	0.020	down
12-Noralliacolide	1.259	1.610	0.020	up
Clofop	1.350	2.711	0.020	up
Adenine monohydrochloride hemihydrate	1.297	1.699	0.020	up

Carpusin	1.278	1.882	0.021	up
3-Methoxy-4-Hydroxyphenylglycol sulfate	1.248	3.010	0.021	up
Acetosyringone	1.240	1.588	0.021	up
Sempervilam	1.243	1.866	0.021	up
Latrunculeic acid	1.272	2.808	0.021	up
Lysinoalanine	1.282	2.189	0.021	up
PE(P-16:0/18:2(9Z,12Z))	1.279	2.435	0.021	up
N-Arachidonoyl Leucine	1.234	0.447	0.021	down
Udp galactose	1.325	2.688	0.021	up
4-Hydroxynonenal glutathione	1.234	1.595	0.021	up
Camphoratin B	1.341	3.357	0.021	up
Lovastatin acid	1.239	1.874	0.021	up
10,16-Dihydroxy-hexadecanoate	1.294	1.520	0.022	up
CPA(18:2(9Z,12Z)/0:0)	1.280	0.428	0.022	down
Urobilin	1.247	2.542	0.022	up
Glaucocalyxin A	1.256	2.360	0.022	up
5-Phenylvaleric acid	1.317	1.941	0.022	up

beta-Isocyclolavandulyl isobutyrate	1.234	2.028	0.022	up
N-Arachidonylglycine	1.402	0.357	0.022	down
Oreskaurin C	1.265	2.700	0.023	up
Tensyuic acid B	1.238	1.539	0.023	up
1-Myristoyl-2-Hydroxy-sn-Glycero-3-Phosphate	1.338	1.738	0.023	up
Decarboxy-Norlobaric Acid	1.482	1.620	0.023	up
Koenimbine	1.270	1.758	0.023	up
2,6-Diamino-9-(2-hydroxyethoxymethyl)purine	1.263	1.504	0.023	up
Salvadione C	1.283	0.358	0.023	down
Triptocalline A	1.226	4.025	0.023	up
N-Palmitoyl Methionine	1.364	0.495	0.023	down
N-Linoleoyl Methionine	1.292	0.496	0.023	down
2-C-methyl-D-erythritol-4-phosphate	1.294	1.531	0.023	up
2-Hydroxytetracosanoic acid	1.334	1.659	0.024	up
Prolyl-Methionine	1.286	1.953	0.024	up
3-hydroxylaurate	1.246	1.752	0.024	up
S-3-oxodecanoyl cysteamine	1.240	2.048	0.025	up

N-Caffeoylputrescine	1.310	1.756	0.025	up
LysoPE(0:0/20:0)	1.404	1.655	0.025	up
PA(12:0/10:0(3-OH))	1.271	2.461	0.025	up
Camphorsulfonic acid	1.253	3.332	0.025	up
N-acetylmethionine	1.444	1.923	0.026	up
Monocillin II	1.242	1.603	0.026	up
3-Trifluoromethyl-4-nitrophenol	1.308	2.898	0.026	up
(ent-6alpha,7alpha)-6,7-Dihydroxy-16-kauren-19-oic acid	1.208	2.392	0.027	up
N-Arachidonoyl Alanine	1.247	0.243	0.027	down
Betaenone F	1.243	2.111	0.027	up
LysoPE(0:0/22:0)	1.366	1.764	0.027	up
Geosmin	1.241	2.163	0.027	up
Palifosfamide	1.242	1.984	0.028	up
Perfluoroheptanoic acid	1.230	1.825	0.028	up
L-2-amino-8-oxodecanoate	1.206	1.721	0.028	up
Irisoquin	1.292	1.855	0.028	up
Tauro-alpha-Muricholic acid	1.215	0.404	0.028	down

PG(18:2(9Z,12Z)/18:2(9Z,12Z))	1.230	2.625	0.028	up
Taurochenodeoxycholic acid	1.231	0.487	0.028	down
Nodakenin	1.257	0.297	0.028	down
Flabellinol	1.207	2.992	0.029	up
N-oleoyl threonine	1.238	0.430	0.029	down
Inositol	1.251	1.539	0.029	up
3,4,6-Trimethoxyphenanthrene	1.195	2.011	0.029	up
Rosarin	1.287	1.579	0.029	up
Perfluorohexanoic acid	1.255	1.961	0.029	up
Galactose 1-phosphate	1.240	1.768	0.029	up
Ixocarpanolide	1.213	2.093	0.030	up
(3R)-hydroxyjuniperic acid	1.281	1.757	0.030	up
Clavaminic acid	1.283	3.051	0.030	up
Acetaminophen glucuronide	1.180	1.754	0.030	up
3-HYDROXYSUBERIC ACID	1.218	1.520	0.030	up
Linalool glucoside	1.330	2.392	0.030	up
7alpha,12alpha-Dihydroxy-3-oxochol-4-enoate	1.334	1.821	0.030	up

Dimethisterone	1.504	13.296	0.031	up
(8E,10S,12Z,15Z)-10-Hydroperoxyoctadeca-8,12,15-trienoate	1.220	2.552	0.031	up
Oleoyl glycine	1.459	0.432	0.031	down
Neobritannilactone A	1.197	1.962	0.031	up
Wilforic acid B	1.183	2.454	0.032	up
Azaserine	1.336	0.549	0.032	down
Citrinin	1.202	1.525	0.032	up
Deoxycholic acid	1.222	2.822	0.032	up
Lamiridosin A	1.193	1.806	0.032	up
Asparaginyl-Tryptophan	1.174	1.529	0.032	up
PTERIN	1.229	2.035	0.032	up
cannabigerolate	1.189	2.323	0.032	up
Epibatidine	1.229	0.202	0.033	down
C18-Sphingosine 1-phosphate; D-erythro-Sphingosine-1-phosphate	1.293	4.252	0.033	up
Flexibolide	1.202	2.930	0.033	up
5,9:6,9-Diepoxy-3-megastigmene	1.177	2.142	0.033	up
Thiolutin	1.210	1.845	0.033	up

4-Methoxy-1,9-cadinadien-3-one	1.273	1.915	0.034	up
Perfluoropentanoic acid	1.229	1.950	0.034	up
2-Heptadecanone	1.283	1.887	0.034	up
3beta,4beta,23-Trihydroxy-24,30-dinor-olean-12,20(29)-dien-28-oic acid	1.212	2.207	0.034	up
Sanaganone	1.344	1.611	0.035	up
PGK1	1.226	2.561	0.035	up
Ile Phe Leu	1.292	2.246	0.035	up
5-(8-Pentadecenyl)-1,3-benzenediol	1.347	0.653	0.035	down
19alpha-19-Hydroxy-3,11-dioxo-12-ursen-28-oic acid	1.181	4.963	0.035	up
Phytocassane E	1.197	1.824	0.036	up
Melatonine	1.168	1.574	0.036	up
Gibberellin A53	1.183	2.126	0.036	up
Saponaceolide E	1.182	2.440	0.037	up
Paeonenoide C	1.188	3.195	0.037	up
1-Stearoyl-sn-glycero-3-phosphate	1.442	1.666	0.037	up
9-epi-solanioic acid	1.172	2.163	0.038	up
1,3-O-di-trans-p-Coumaroylglycerol	1.180	2.055	0.038	up

10-EdAM	1.168	1.686	0.038	up
ent-19-Trachylobanal	1.168	0.665	0.039	down
(plusmn)9-HpODE	1.237	1.671	0.039	up
17beta-Hydroxy-4-oxa-5alpha-estr-1-en-3-one acetate	1.139	2.702	0.039	up
(2S,4S)-Monatin	1.171	1.633	0.039	up
Nubenolide acetate	1.167	2.185	0.039	up
Asparaginylglutamine	1.144	1.906	0.040	up
Tridecanoylglycine	1.244	2.319	0.040	up
2-Undecanone	1.245	2.325	0.040	up
(5Z,8Z)-tetradecadienoylcarnitine	1.188	0.513	0.040	down
Steviol	1.246	0.643	0.041	down
2-Thiouridine	1.238	2.647	0.041	up
Carboxyibuprofen	1.232	1.598	0.041	up
Homaline	1.152	1.613	0.041	up
2-[4-(3,4-Methylenedioxyphenyl)butyl]-4(1H)-quinolinone	1.143	1.743	0.042	up
Sinapoyltartronate	1.252	4.419	0.042	up
Cytochalasin IV	1.348	1.575	0.042	up

N-Palmitoyl Valine	1.136	0.451	0.042	down
Schidigeragenin B	1.184	0.327	0.043	down
Homochlorcyclizine	1.139	2.799	0.043	up
Cadusafos	1.176	2.003	0.044	up
PA(12:0(3-OH)/10:0(3-OH))	1.238	3.073	0.044	up
7-Hydroxymethyl-12-methylbenz[a]anthracene sulfate	1.227	2.364	0.044	up
2,5-Diethyltetrahydrofuran	1.146	1.814	0.045	up
7-Oxo-8,15-isopimaradien-18-oic acid	1.152	1.626	0.045	up
Ebeietinone	1.130	0.367	0.045	down
Cyclic ADP-ribose	1.131	2.130	0.045	up
Chlamydocin	1.204	0.309	0.045	down
(3R,5R)-3-(((3R,5R)-3,5-dihydroxy decanoyl)oxy)-5-hydroxydecanoic acid	1.145	1.515	0.045	up
Ixerisoside I	1.154	0.533	0.045	down
Besarhanamide A	1.167	0.428	0.046	down
6-Chloromelatonin	1.202	1.675	0.046	up
N-Linoleoyl Valine	1.135	0.520	0.046	down
Tortifolisine	1.134	0.476	0.046	down

Alpha-Muricholic acid	1.126	1.924	0.046	up
1,25-Dihydroxy-19-norvitamin D3	1.207	0.418	0.046	down
Enzastaurin	1.176	0.247	0.046	down
Hypochoeroside K	1.214	1.925	0.047	up
3-Thiatetradecanoic acid	1.145	2.464	0.047	up
2-Pentyloxazole	1.170	1.659	0.047	up
S-(3-Methyl-2-butenyl) 2-methylpropanethioate	1.168	2.126	0.047	up
1-Dodecanesulfonic acid, 1-hydroxy-3-oxo-	1.197	1.535	0.048	up
Biotin thiamine	1.197	2.195	0.048	up
3alpha-Hydroxyayapanonic acid	1.175	1.670	0.048	up
3Beta-Hydroxy-23,24-Bisnorchol-5-Enic Acid	1.189	0.641	0.049	down
Guanosine-5'-diphospho-beta-L-fucose	1.132	2.307	0.049	up
8'-O-methylasterric acid	1.141	1.861	0.049	up
D-Glucose 6-sulfate	1.193	2.484	0.049	up
Pentamethoxyacetophenone	1.161	3.455	0.049	up
Glu Ile Leu	1.102	1.530	0.049	up
Epoxycytochalasin H	1.150	2.366	0.050	up
